# Validation of an MD simulation approach for electrical field responsive micelles and their application in drug delivery

**DOI:** 10.1038/s41598-023-29835-y

**Published:** 2023-02-15

**Authors:** Leila Razavi, Heidar Raissi, Farzaneh Farzad

**Affiliations:** Department of Chemistry, University of Birjand, Birjand, Iran

**Keywords:** Biochemistry, Drug discovery

## Abstract

In the current work, a new type of micelle is designed that has active connectivity in respond to exterior stimulus and the desired water solubility. Two end-ornamented homopolymers, polystyrene-beta-cyclodextrin (PS-β-CD) and polyethylene oxide-ferrocene (PE-FE), can aggregate as a supramolecular micelle (PS-β-CD/PE-FE) by the guest–host interactions. Our results showed that the Lennard–Jones and hydrophobic interactions are the main powerful forces for the micelle formation process. It was found that the electrical field plays a role as a driving force in the reversible assembly-disassembly of the micellar system. Moreover, for the first time, we examined the PS-β-CD/PE-FE micelle interaction as a drug delivery system with anastrozole (ANS) and mitomycin C (MIC) anti-cancer drugs. The investigation of the total energy between PS-β-CD/PE-FE micelle and drugs predicts the drug adsorption process as favorable (E_total_ = − 638.67 and − 259.80 kJ/mol for the Micelle@ANS and Micelle@MIC complexes, respectively). Our results offer a deep understanding of the micelle formation process, the electrical field-respond, and drug adsorption behaviors of the micelle. This simulation study has been accomplished by employing classical molecular dynamics calculation.

## Introduction

Nowadays, modern nano-scale systems for nanomedicine applications, especially in the field of drug delivery, have received a lot of attention. The effectiveness of anti-cancer drugs is often reduced by non-dedicated distribution in cells and tissues, some of these drugs are quickly metabolized or excreted from the body. Hence, it is essential to use nano-carriers that correctly target diseased sites in the body. Due to the remarkable progress in science materials and pharmaceuticals, a wide variety of nano-carriers with different sizes, architectures, and surface properties have been used. Among these polymer nanoparticles, metal oxide frameworks, dendrimers, liposomes, and micelles can be mentioned^1,2^. Combining drugs in micellar assemblies is an appropriate way to raise their therapeutic index and solubility^3,4^. The obtained experimental results illuminate that optimizing the function and structure of polymeric micelles gives a good formula for developing supramolecular devices sensitive to the intracellular environment for cancer therapeutic applications and resistant cancer treatment with limited vasculature. Relatively small size and the possibility of making micelles on a large scale are their two unmatched advantages compared with other drug delivery strategies. Thus, the micellar system with a small size could substantially ameliorate the performance of encapsulated drug molecules in the body. This enables simple drug formulation and ease of manufacturing; other advanced drug delivery systems (DDSs) are complicated to prepare and have inherent problems that hinder their large-scale production stably and consistently. These two advantages of micelles have spurred their acceptance as a first-line drug formulation technology^5,6^.

Anastrozole (ANS, Fig. S1A) is a well-known anti-cancer drug in the treatment of cancer in the breast^7–9^. ANS is an aromatase inhibitor that decreases the overall estrogen levels, also this inhibitor blocks the conversion of androgens to estrogen. The serious side effects of ANC drug include high blood pressure, hot flashes, unitability in circulation, etc.^10,11^.

Mitomycin C (MIC, Fig. S1B) is a DNA-alkylating factor, that has received wide consideration in different disease research areas, particularly in cancer treatment^12–14^. Clinically, it has been utilized for the treatment of various cancers such as colorectal cancer, lung cancer, pancreatic cancer, gastric carcinoma, etc.^15^. Though MIC shows an excellent antitumor effect, its intravenous administration must be conducted attentively to avert extravascular leak at the injection location. Also, the catheter site must be attentively monitored to avoid possible necrosis due to the high toxicity caused by the non-specific DNA-alkylating ability. All these shortcomings greatly limit the further use of ANC and MIC in cancer treatment. Therefore, in this study, we have proposed an appropriate DDS to prevail over such restrictions. The bioavailability of drugs refers to the extent and rate at which the active drug enters systemic circulation, thereby accessing the site of action, and depends partly on its design and manufacture. The poor bioavailability of drugs is mainly attributed to the poor aqueous/lipid solubility and low plasma membrane permeability^16,17^.

Stimulus- respond systems formed of smart polymers can under chemical or physical modifications, such as changes in size and volume, in response to external stimulus. As a smart assembly, they can form their aggregated structures in respond to an external stimulus such as temperature^18–22^, pH^23–25^, redox^26–29^, and so on. The guest–host interplay is also a non-covalent interaction for the fabrication of such systems, for instance, beta-cyclodextrin (β-CD) can aggregate with ferrocene (FE). Typically, uncharged FE species or their derivatives bind strongly in the β-CD cavity, while the charged species (FE^+^) detached quickly from the cavity. It has been observed that this process can be reversibly changed under an exterior stimulus such as applying an external EF^30^. To understand the EF-respond polymer micelles, polystyrene with β-CD end-ornamented (PS-β-CD) and polyethylene oxide with FE uncharged end-capping (PE-FE) are modeled, and their micellization behavior is investigated in detail. This supramolecular copolymer composed via guest–host interaction is expected to be reversible under the effect of external EF, that induces an assembly-disassembly of the micelle. Several research works have shown that artificial electrical field (EF)- respond micelles are ideally suited for the adsorption of drugs and controlled release^31–33^. EF-respond micelles are useful for oral drug delivery because they can prevent the release of drugs and precipitation after dilution in the upper gastrointestinal tract. After oral administration and in weak EFs, these drug-loaded micelles will remain in the self-assembled form at the stomach and reduces drug release. At higher EF, the self-assembled systems in the small intestine begin to dissociate, resulting in the release of the entrapped drug in a molecularly dispersed form. This drug release type from the EF-sensitive systems will offer progressive drug dissolution in the gastrointestinal tract and also maximize its release in the small intestine, which provides the largest point for drug adsorption. This can be useful for the oral bioavailability of the combined drug by increasing its carry and transportation across the gut wall into circulation. Therefore, it is interesting to investigate the EF-sensitive micelle's potential to improve the oral bioavailability of poorly water-soluble drugs^34^.

Molecular dynamics (MD) simulation is a useful and accurate computational tool, widely approved and accepted as a reliable simulation in chemistry. This method has progressed sufficiently to allow the detailed investigation of complex molecular systems that are often inaccessible to experiments. So that the MD calculation has been widely used to investigate micellar systems and obtain dynamic and structural properties. Through the MD method can also realize the biological functions of biological macromolecules, the interaction mechanism between small molecules-proteins, and the self-assembly process of nanomaterial molecules at the molecular level. In the drug adsorption process into micelles, a theoretic approach based on molecular simulation can also constitute a beneficial tool that decreases cost and time and supports experimental research^35,36^. For instance, Luo et al.^37^ conducted an MD simulation to investigate the aggregation/de-aggregation of the pH-sensitive copolymer, composed of poly (β-amino ester) and methyl ether-capped (polyethylene glycol). Besides, they show that the poly (β-amino ester)/methyl ether-capped (polyethylene glycol) micelle is an appropriate candidate for the loading/releasing of the camptothecin drug.

Herein, we examined an EF-respond supramolecular micelle based on the aggregation of two homopolymers, namely, PS-β-CD/PE-FE using the MD simulation method. Through the host–guest interactions between PS-β-CD and PE-FE, two molecules connect, constituting a non-covalent micelle. Furthermore, by applying an external EF, a reversible disassembly/assembly transition of this micellar complex came true. As well as, the adsorption of ANS and MIC anticancer drugs based on this complex is also conducted successfully. Generally, our aim is to validation of an MD Simulation approach for EF-sensitive micelles based on the orthogonal assembly of two homopolymers and their application in drug delivery.

## Materials and methods

### Systems explored

**Figure 1:**
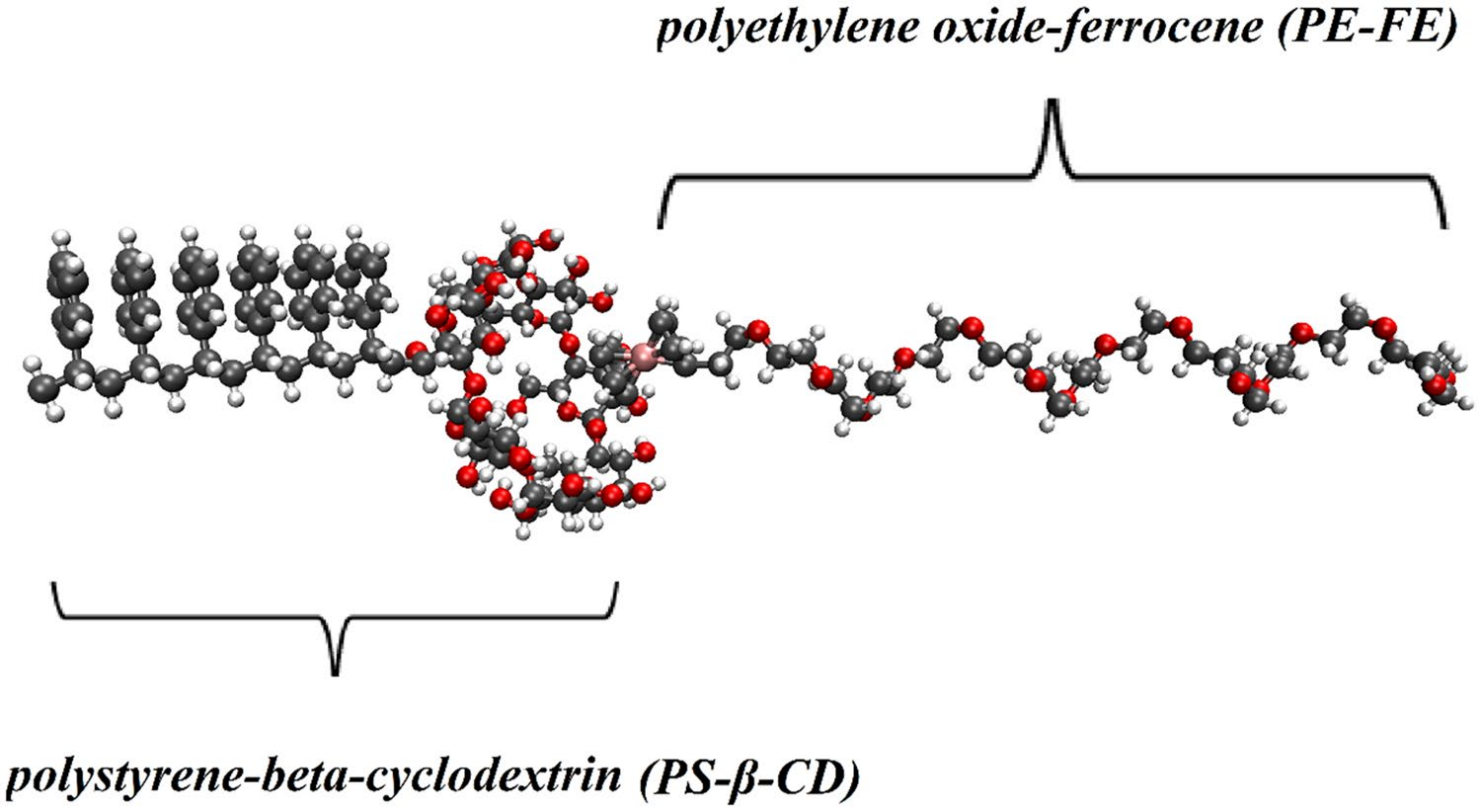
Structure of PS-β-CD and PE-FE molecules.

**Figure 2:**
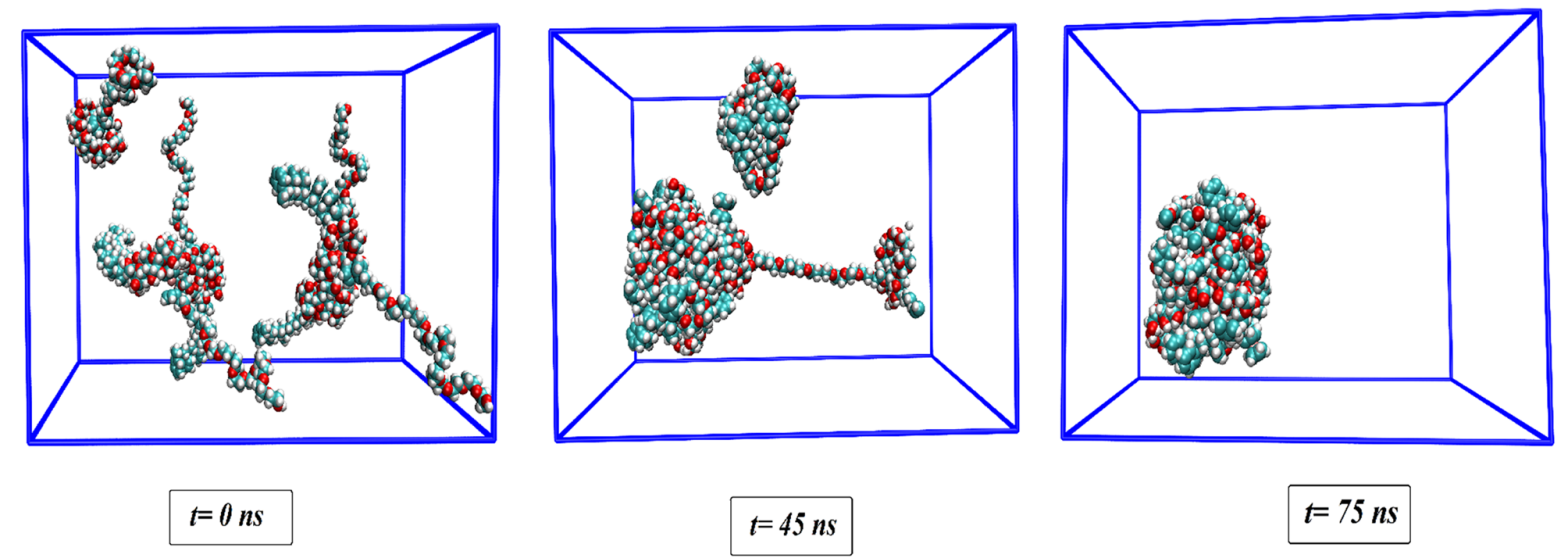
Trajectory snapshots of PS-β-CD/PE-FE aggregation. The MD trajectories are further viewed and analyzed with VMD (ver 1.9.3) and GROMACS tools (https://www.ks.uiuc.edu/Research/vmd and http://www.gromacs.org).

The beta-cyclodextrin, ferrocene, and two polymers (i.e., PS and PE) structures are generated using GaussView software (see Fig. S2)^38^. The molecular structure of MIC and ANS drug molecules is obtained from the PubChem database and more information about these two drugs is given in Table S1. We designed two biocompatible homopolymers as the end coupling linkers, (Micelle system): one is the PS homopolymer modified with β-CD (PS-β-CD), and another is a PE homopolymer modified with FE (PE-FE) (Fig. 1). The two homopolymers will connect via host–guest interactions between β-CD and FE to compose a non-covalent supramolecular block copolymer (PS-β-CD/PE-FE). PS-β-CD and PE-FE can self-assemble in an aqueous solution to constitute supramolecular micelles as shown in Fig. 2. By applying an EF, the reversible assembly-disassembly transition of PS-β-CD/PE-FE micelle is realized) more details are in Table S2(. Also, two systems have been designed to ascertain the PS-β-CD/PE-FE capability as a drug carrier. In these simulation boxes, ANS and MIC molecules are distributed approximately at an initial distance of ~ 2 nm from the micelle, which are named the Micelle/ANS and Micelle/MIC systems, respectively. It should be noted that the final structure of the self-assembled PS-β-CD/PE-FE complex has been extracted as a carrier. Further details about the simulation boxes can be found in Table S3 The dimensions of the simulation box used for all the systems are 6 × 6 × 9 nm^3^.

### Simulation details

The GROMACS 5.1.4 package (see http://www.gromacs.org) to perform MD simulations with the CHARMM force field is utilized^39^. The force field parameters for the beta-cyclodextrin, ferrocene, polymers, and drugs are obtained from the CHARMM36 force field^40^. Each box simulation is solvated in TIP3P water, and any net charge is neutralized by adding an appropriate number of Na^+^ and Cl^−^ ions^41^. Temperature is constated by applying the V-rescale thermostat (T = 310 K), and pressure is retained with Berendsen barostat (P = 1 bar)^42–44^. The leap-frog algorithm is utilized to solve Newton’s equations of motion with a time-step of 2 fs time step^45^. A cutoff radius of 1.4 nm is adopted for nonbonded interactions, and long-range electrostatic interactions are assessed with the particle mesh Ewald method^46^. Vibrations of bonds containing hydrogen atoms are constrained via the Linear Constraint Solver algorithm^47^. The total simulation time is 75 ns for each simulation run. The MD trajectory is further viewed and analyzed with visual molecular dynamics (VMD, ver 1.9.3) and GROMACS tools^48^. To calculate the critical aggregation concentration (CAC) in physical units (mg/ml), the Eq. (1) can be used, enabling a comparison to experimental values^49^:

1 $$\mathrm{CAC}=\frac{n}{{L}^{3 }{N}_{A}}$$

where, n is the number of surfactant molecules in the box, L is the side length of the simulation box, and N_A_ is Avogadro’s number.

#### Radial distribution function (RDF, g(r))

One of the most important analyses regarding micelles formation is the distribution study of molecules such as drugs around these structures and is determined as the following statement^50^.

2 $$\mathrm{gij}(\mathrm{r}) =\frac{<{\uprho }_{j }(r)>}{{{<\uprho }_{j }>}_{local}}$$

where, $<{\uprho }_{j }\left(r\right)>$ is the particle density of type j at a distance r around particles i, and ${{<\uprho }_{j }>}_{local}$ corresponds with the particle density of type j averaged over all spheres around particles i with radius r_max_.

#### The number of hydrogen bonds

Based on the geometric criterion as follows, hydrogen bond analysis shows the number of hydrogen bonds formed between the donor and the acceptor.

3 $$\mathrm{r}\le {\mathrm{r}}_{\mathrm{hydrogen\, bond}}=0.35 \, \, \mathrm{ and} \, \, \alpha \le {\alpha }_{\mathrm{hydrogen\, bond}}= 30 {^{\circ}}$$

The number of hydrogen bonds created between the simulated components over time provides insight into the forces nature that exists between them^51^.

#### The mean-square displacement (MSD)

The MSD and self-diffusion coefficient (D_i_) are considered to evaluate the molecule's mobility^52,53^, the definition of MSD can be found in our previous study^54^. The following equation is applied to appraise the MSD^55,56^:

4 $${\text{MSD }}\left( {\Delta {\text{t}}} \right) \, = \, < \, \left( {{\text{r}}_{{\text{i}}} \left( {\Delta {\text{t}}} \right) \, - {\text{r}}_{{\text{i}}} \left( 0 \right)} \right)^{{2}} > \, = \, < \, \Delta {\text{r}}_{{\text{i}}} \left( {\Delta {\text{t}}} \right)^{{2}} >$$

where r_i_ (Δt) − r_i_ (0) is defined as the distance traveled by COM of the particle i over some time interval of length.

Theoretically, the D_i_ is accurately computed based on the Einstein equation and practically could estimate the D_i_ from the slope of the MSD versus time.

## Results and discussion

### The Micelle formation process

To explore the self-assembly process of PS-β-CD/PE-FE micelle, a simulation box containing 5 PS-β-CD/PE-FE molecules is designed (Fig. 2, 0 ns). As depicted in this figure, with a random distribution of PS-β-CD/PE-FE molecules, the aggregation of these molecules is observed to be at 45 ns. The oligomers then come closer together to constitute a larger oligomeric cluster reaching the lowest energy level. After this time, the constituted micelle remains stable until the simulation ends (75 ns). Total energies (Lennard–Jones (L–J) + Coulombic (Coul)) of PS-β-CD/PE-FEs, as well as their interactions with water at the beginning and end of the MD simulation, are calculated (Tables 1 and Tables S4). The interaction between water and PS-β-CD/PE-FE molecules decreases considerably in the self-assembly process of PS-β-CD/PE-FE. This behavior illustrates that the number of water molecules surrounding PS-β-CD/PE-FE molecules are directed around it and confirms the aggregation behavior of PS-β-CD/PE-FE micelle. In other words, it can be seen, by increasing the interactions of PS-β-CD/PE-FE molecules, their interactions with water are decreased. The CAC value obtained for Micelle system is 0.198 mg/mL (~ 0.2 mg/mL) in this study which is in good agreement with the experimental data reported by Yan et al.^30^. We also found that aggregates' size formed by PS-β-CD/PE-FE molecules raise over time (about 13 Å to 53 Å), which can be verified by plotting the size of aggregates as a function of simulation time, as shown in Fig. S3.

**Table 1:** The L–J, Coul and total interaction energies between PS-β-CD/PE-FE molecules in the investigated systems (all energy values are in kJ/mol).

Complexes	L–J	Coul	Total
Micelle	− 3751.54	− 1487.34	− 5238.88
Micelle (+ 1.5)	− 3770.1	− 1316.96	− 5087.06
Micelle (+ 3)	− 1834.66	− 1760.19	− 3594.85
Micelle (− 3)	− 4243.13	− 1552.28	− 5795.41

### EF-respond behavior of PS-β-CD/PE-FE micelle

**Figure 3:**
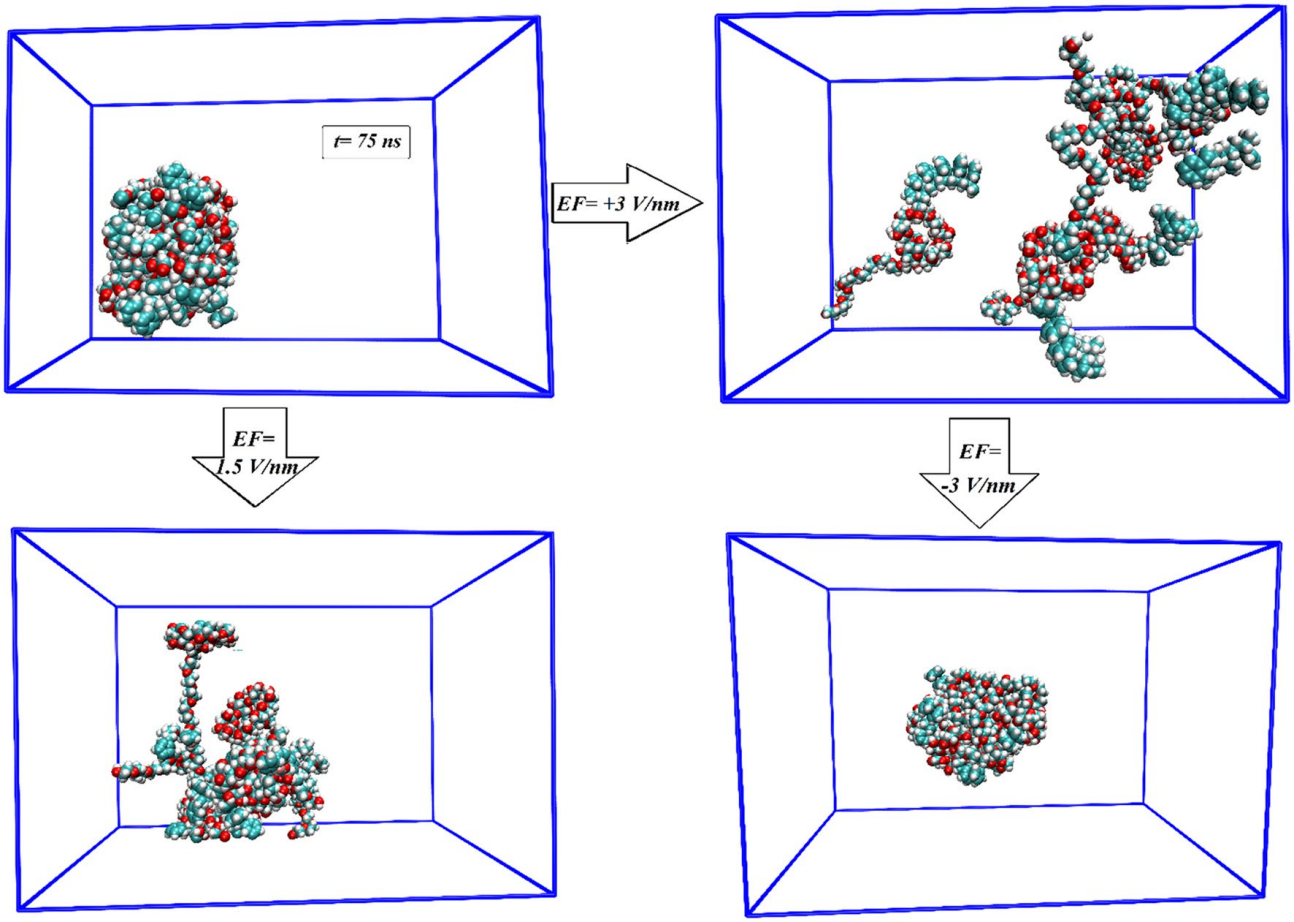
EF-responsive controlled assembly and disassembly of PS-β-CD/PE-FE supramolecular micelle.

In this section, by applying external electric fields, a reversible assembly-disassembly process of the micellar system is investigated. For this purpose, the PS-β-CD/PE-FE micelle is subjected to external EFs with an electric field strength of + 1.5 (Micelle (1.5) system) and 3 V/nm (Micelle (+ 3) system). The external EF with various strengths is exerted in three directions (x, y, and z), and the computed energies in these systems are given in Table 1. The decrease in the values of total energy in the Micelle (1.5) and Micelle (+ 3) systems shows that the EF acts as a driving force in the reversible assembly-disassembly of the micellar system. As seen in Fig. 3, when no stimulus is applied, the PS-β-CD/PE-FE micelle has a nearly perfect spherical shape. After exerting an EF with the electric field strength of + 1.5 V/nm, the micelle began to decompose, and the micelle structure is partially disassembled. When the EF raised from + 1.5 to + 3 V/nm, it is seen that the micelle could entirely self-disaggregate into small piecesss within 75 ns, denoting the whole disassembly of the micelle structure (Fig. 3, EF =  + 3 V/nm). Also, it is worth mentioning that there is a reduction in the total energy value in the Micelle (+ 3) system with increasing field strength. The disassembly system can be reassembled by applying a reductive EF with a negative electric field of − 3 V/nm, and micelles with similar sizes and shapes can be reformed. After exerting a negative EF of − 3 V/nm, the micelle reformed and recovered its spherical shape because the Fe (Fe atom in the PE-FE component) loses one electron and changed to PE-FE^+^ which can interact with PS-β-CD again [Micelle (− 3) system, (Fig. 3, EF = − 3 V/nm)]. This fact is confirmed by the reduction of the total energy in the Micelle (− 3) system (see Table 1). Close inspection of snapshots also confirms that the EF-respond ability of the PS-β-CD/PE-FE assembly is reversible.

**Figure 4:**
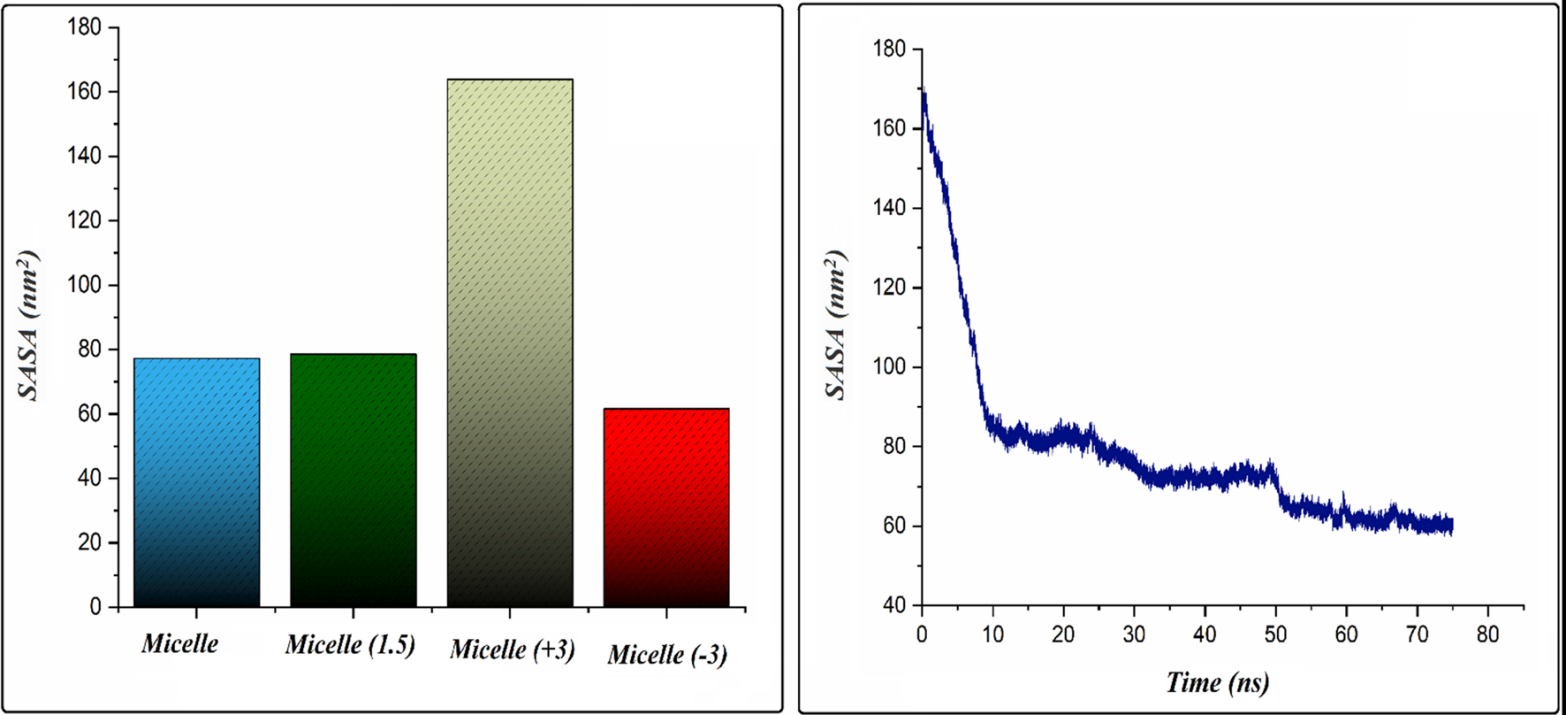
Average value of the solvent accessible surface area (SASA) for PS-β-CD/PE-FE molecules in the investigated systems (left) and the variations of SASA in Micelle system as a function of simulation time (right).

The solvent accessible surface area (SASA) for the three mentioned above systems is shown in Fig. 4. The SASA is the surface area of compositions that is accessible to contact with solvent molecules^53^. The SASA plot of the Micelle system decreases rapidly upon a few nanoseconds after starting the simulation as the aggregation of PS-β-CD/PE-FE molecules commences and then preserves an average value of 61.02 nm^2^ (Fig. 4, right). The decrease in SASA of the micelle is because the aggregation of PS-β-CD/PE-FE molecules in the solution prevents their contact with the water molecules. Furthermore, the higher values of SASA for the Micelle (+ 3) system in comparison to the other systems confirms that this system has more surface area, which is available to interact with water molecules.

**Figure 5:**
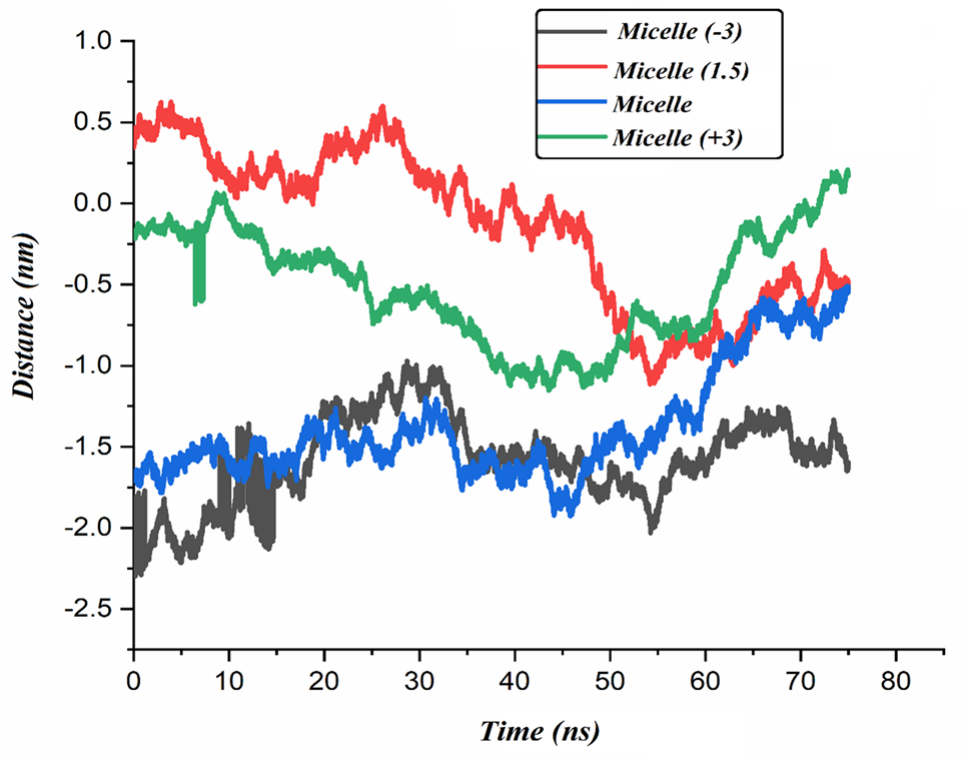
The average center-of-mass (COM) distance between the PS-β-CD/PE-FE molecules during of simulation.

To further investigate the interaction between PS-β-CD/PE-FE molecules, the distance between the center of mass (COM) of these molecules is calculated (Fig. 5). Determination of the monomer–monomer distance in the micellar aggregates is important to a detailed description of the general aggregation/disaggregation process. The number of hydrogen bonds, hydrophobic interactions and decreases electrostatic repulsion play key roles in decreasing the distance between PS-β-CD/PE-FE molecules and the process of micelle formation. In the Micelle (+ 3) system, the distance plot shows that the distance between the PS-β-CD/PE-FE molecules in this system is greater than in the others. This result confirms the effect of external EF in the reversibility of assembly-disassembly of the micellar system.

Understanding the molecular mechanism of EF-respond reversible assembly-disassembly micelles can greatly contribute to realizing an ideal DDS with both high delivery efficacy and less damage to the cell. indeed, responsive systems will open up new directions toward controlled drug release carriers and new therapies. Experimental studies have reported that by applying an EF, nanoparticles can directly permeate across the cell membrane without the confinement of nanoparticles by endocytic vesicles, while damage to the cell can often be a concern^1,57,58^.

### The capability of PS-β-CD/PEO-FE micelle as a drug delivery platform

#### Investigating the equilibration

**Figure 6:**
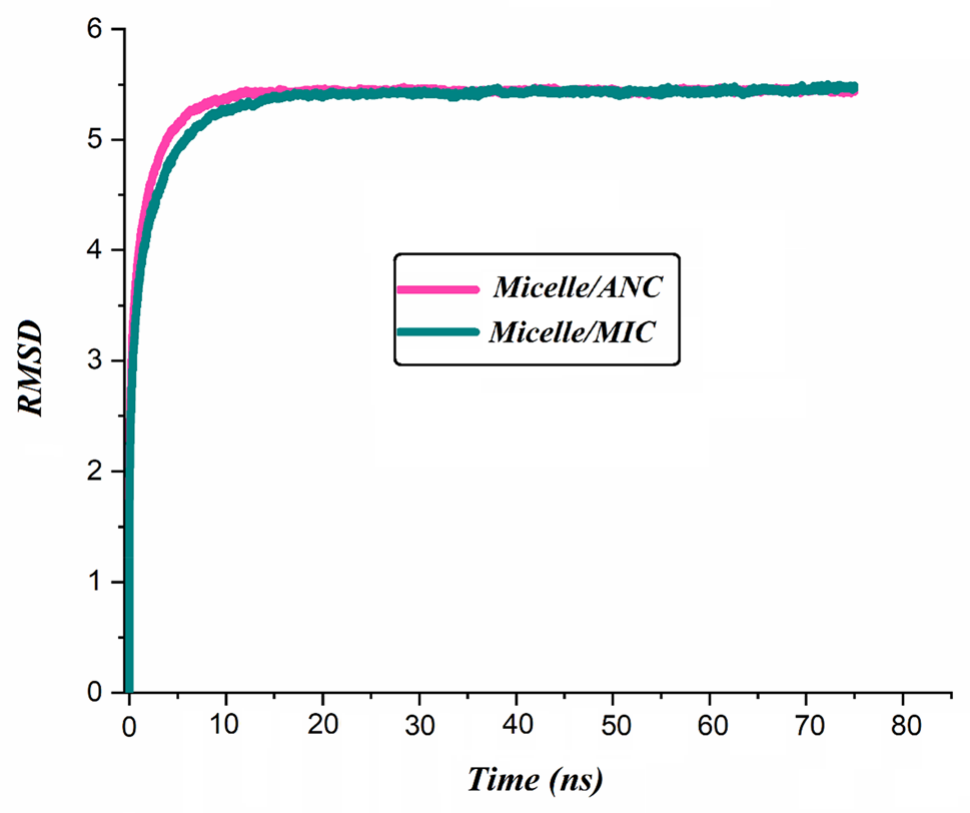
RMSD versus time of PS-β-CD/PE-FE micelle and drug molecules in 75 ns simulation.

At first, the root means square deviation (RMSD) is calculated to ensure that the PS-β-CD/PE-FE@drug complexes remained stable during the simulation (Fig. 6). The curve of the RMSD of the system also remained stable after ~ 12 ns, which demonstrated the system quickly achieved equilibrium in this simulation. Having confirmed the stability of drugs in our model, details of the adsorption mechanism of ANS and MIC on PS-β-CD/PEO-FE micelle are presented below.

#### Loading of the ANS and MIC molecules on the PS-β-CD/PE-FE

**Figure 7:**
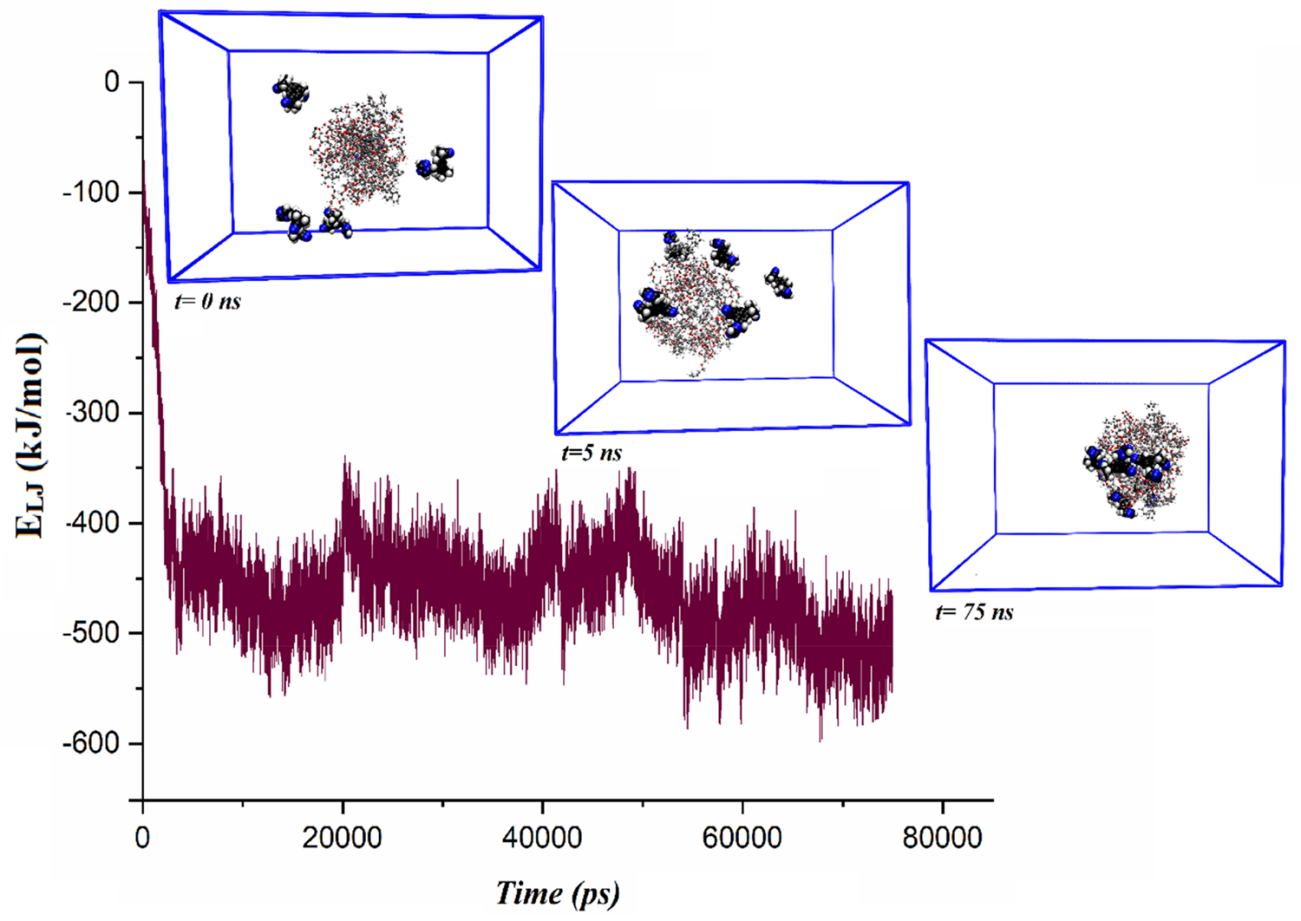
The L–J energy variations in ANS-loading process.

**Figure 8:**
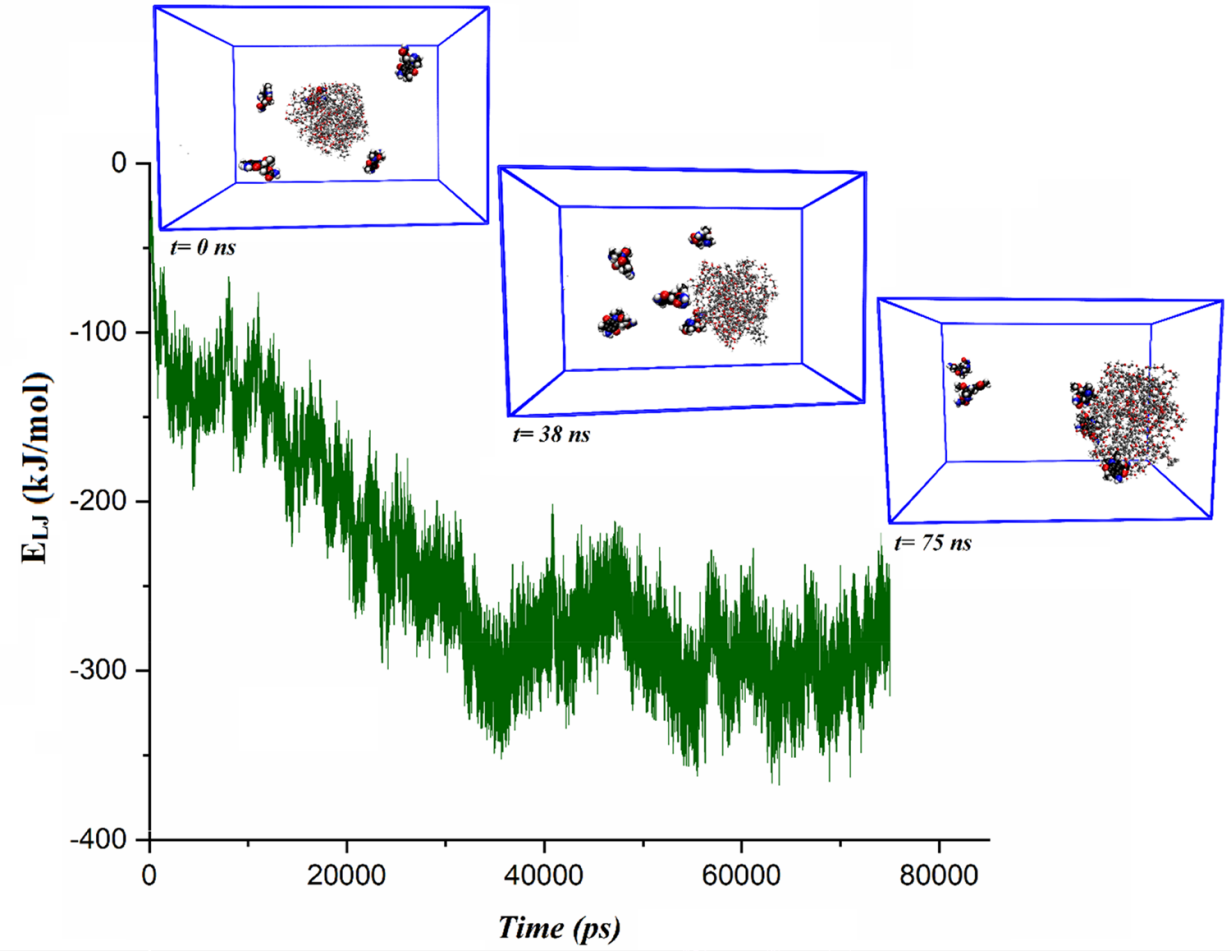
The L–J energy variations in MIC-loading process.

The loading trend of ANS and MIC molecules onto the PS-β-CD/PE-FE micelle is shown in Figs. 7 and 8. As is observed in these figures, at first, 5 drug molecules are distributed randomly around the PS-β-CD/PE-FE micelle. As the adsorption proceeds, the distance between the micelle with drug molecules decreases and approaches its minimum value at equilibrium. Finally, the PS-β-CD/PE-FE micelle spontaneously encapsulates ANS molecules and forms near-spherical shape micelles (Fig. 7, 75 ns). Comparison Figs. 2 and 7 exhibit that the formation process of the ANS-loaded micelles is similar to the blank micelles, and finally, a single spherical micelle is formed. In the Micelle/MIC system, it is observed that just 60% MIC is adsorbed into the micelles, leaving a few numbers exposed on the outside surface of the micelle, suggesting a decrease in its MIC-loading capacity (see Fig. 8).

#### Drugs-micelles interactions

To quantitatively investigate the interaction of ANS and MIC to the micelle, the total energy between the PS-β-CD/PE-FE and drugs is calculated and reported in Table 2. Corresponding to this table, the Coul average energy is considerably less than the calculated average L–J energy between the PS-β-CD/PE-FE micelle and drug molecules. This result proves that L–J interactions are predominant during the drug adsorption process and the Coul interactions also play an important role during adsorption. This fact is due to the PS-β-CD/PE-FE molecules can easily form hydrogen bonds with ANA and MIC molecules. Furthermore, the total energy of MIC molecules in the Micelle/MIC system becomes less negative compared to Micelle/ANS system. This is in line with presented snapshots in Fig. 8, 75 ns, in which it is shown that two MIC molecule is located further away from the micelle, and three other MIC are adsorbed onto the surface of the micelle. In the Micelle/ANS system all ANS molecules are trapped inside the micelle, hence having high total energy. To further clarify this issue, changes in L–J energy vs. time are also depicted in Figs. 7 and 8, indicating when loading the drug molecules onto the micelle (t ~ 3. and 62 ns for Micelle/ANS system and t ~ 10 and 60 ns for Micelle/MIC system), the L–J interaction energy becomes more and more negative. Upon the Micelle@ANS complex’s formation in ~ 16 ns, it reaches an energy equilibrium state of − 700 kJ/mol, which is about − 100 kJ/mol more negative than the Micelle/MIC system.

**Table 2:** The obtained pair interaction energy of drug molecules with micelle in the drug delivery systems (All energy values are in kJ/mol).

Complexes	L–J	Coul	Total
Micelle/ANS	− 457.99	− 180.68	− 638.67
Micelle/MIC	− 242.48	− 17.32	− 259.80

#### RDF analysis

**Figure 9:**
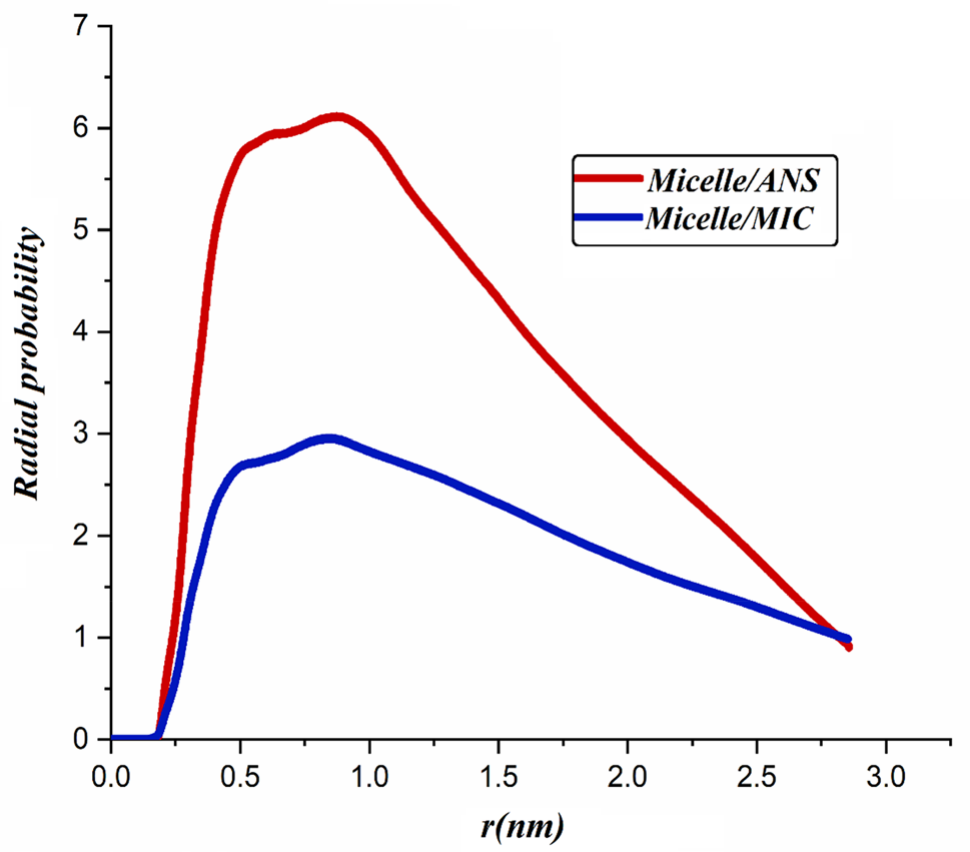
ANS and MIC molecules distribution from the COM of micelle.

The results of the RDF analysis between the micelle and the molecules of drug are illustrated in Fig. 9. As it is obvious from this figure at a short distance (~ 0–0.16 nm), radial probability (g(r)) is zero as a result of the strong repulsive of carrier-drug pairs^59^. Concerning the RDF diagram of the micelle-drugs model in Fig. 9, the maximum distribution of ANS and MIC molecules lies in the range of ~ 0.3 to 1.3 nm. This condition indicates that the ANS and MIC molecules are concentrated around the PS-β-CD/PE-FE micelle. The RDF plot for both examined systems indicates one sharp peak at a distance of ~ 0.8 nm, which is relevant to hydrogen bonds. Also, according to the obtained results, it is seen that the probability of finding ANS molecules around the PS-β-CD/PE-FE is considerably more than the MIC molecules. This fact can be attributed to the stronger interaction of ANS molecules with the micelle. To further advocate this micelle-drugs interaction trend, water-micelle (in the Micelle system) and water-drugs RDFs are computed and depicted in Fig. S4. As expected, the height of the water molecule's RDF peak around micelle and drug molecules is less than the micelle-drugs RDF peak. As a result, water distribution around micelle and drugs indicates that PS-β-CD/PE-FE molecules have hydrophobic properties. It is worth noting that the mentioned RDF trends correspond to the energy results in the previous section.

#### The number of hydrogen bonds

**Figure 10:**
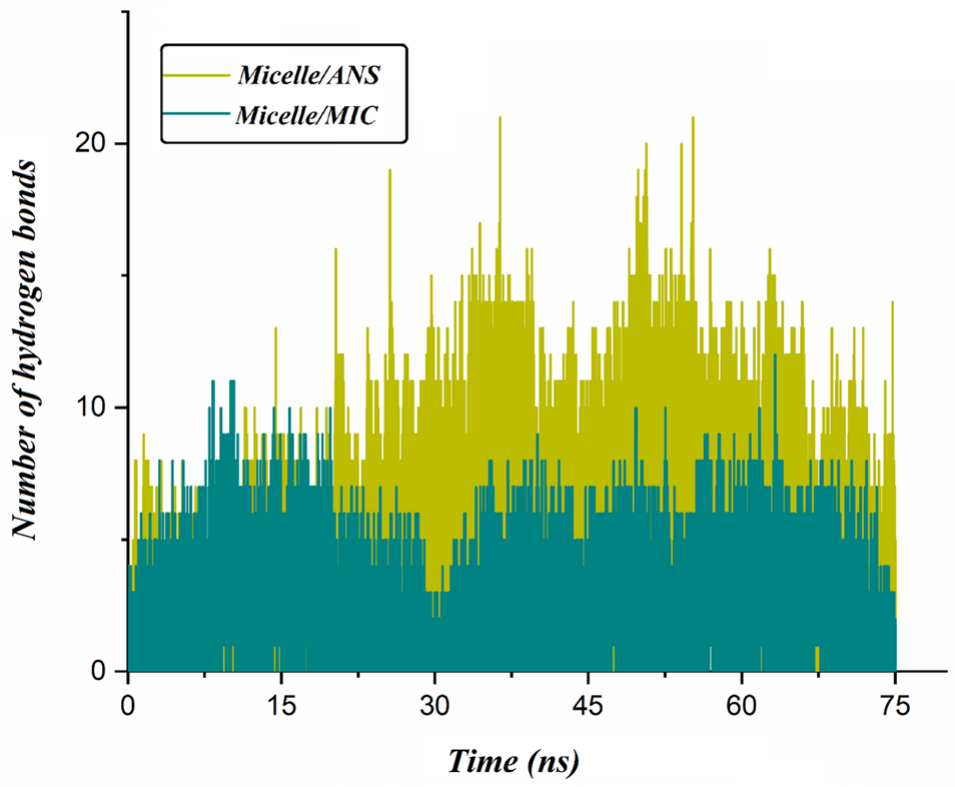
The number of hydrogen bonds between micelle and drug molecules as a function of time.

The hydrogen bond changes between micelles and drug molecules are shown in Fig. 10. In both of the investigated systems, the number of hydrogen bonds is raised; this increment has a direct role in the enhancement of their binding affinity. Close inspection of Fig. 10 shows that the average number of hydrogen bonds for the Micelle/ANS system is more than the Micelle/MIC system. This result is likely due to an raised number of ANS molecules at the micelle surface.

#### MSD analysis

**Figure 11:**
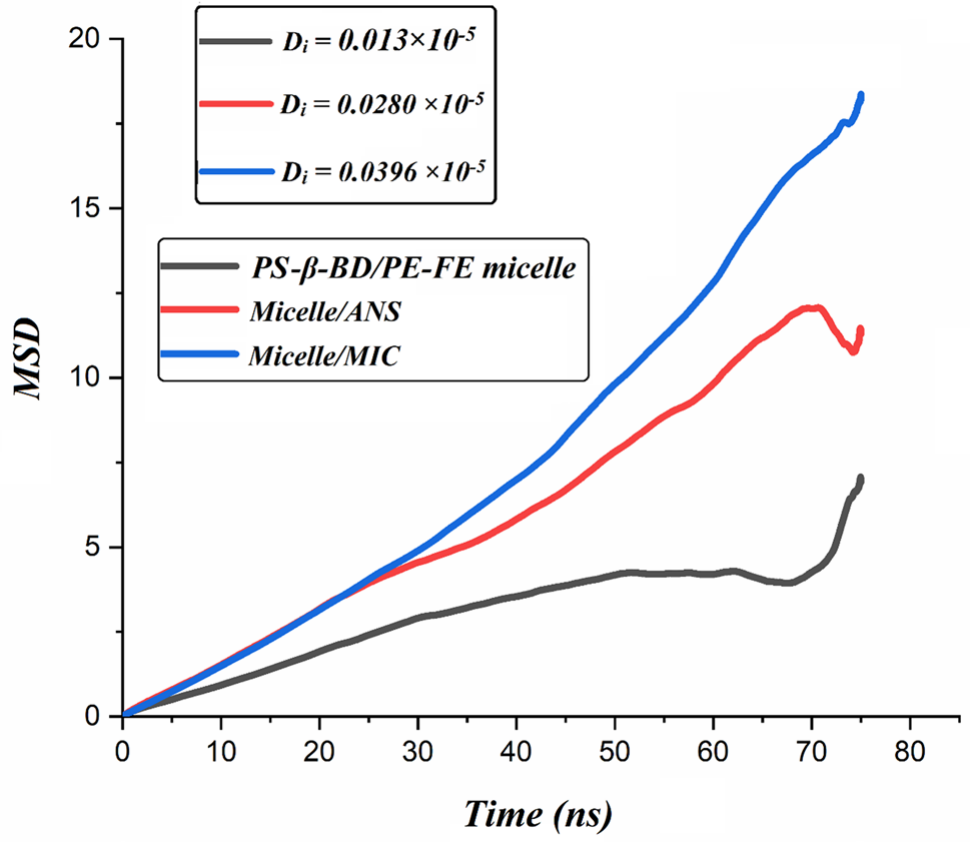
Time evolutions of MSD in the investigation systems.

Figure 11 illustrates values of D_i_ and MSD plots for micelle and drug molecules as a function of simulation time. As it is obvious from this figure, the slope of the MSD plots changes upon drug adsorption. In the Micelle/ANS system, the movement of ANS molecules becomes slower upon the drug is adsorbed into the micelle. Close inspection of Fig. 11 demonstrates that compared to the freely moving drug molecules, the mobility of PS-β-CD/PE-FE micelle is limited. The D_i_ for ANS molecules is lower than MIC, which indicates the total energy of ANS is more, and its adsorption on the PS-β-CD/PE-FE is faster than the MIC. Furthermore, the lower value is related to the slower release of ANS from the micelle and consequently, the relatively high drug loading capacity. In other words, ANS drug is less diffusive compared to MIC which means it would be sustained in the micellar system for a longer time.

#### COM distance

**Figure 12:**
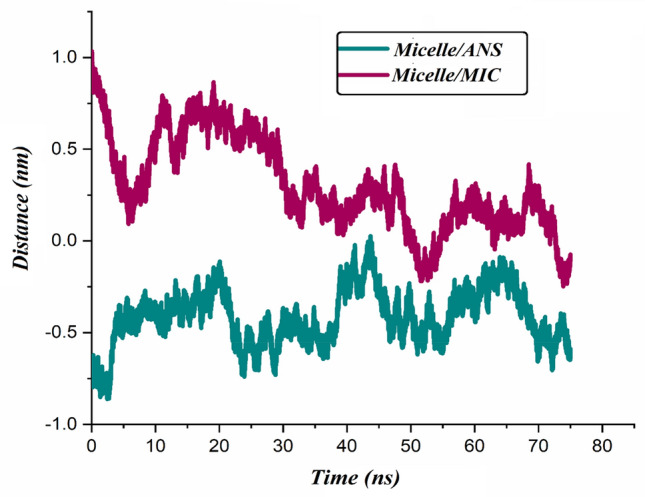
The average COM distance between micelle and drug molecules during of simulation.

In order to inspect the interaction of micelle with drugs, the COM distance between the PS-β-CD/PE-FE complex and drug molecules as a function of the simulation time is calculated and given in Fig. 12. As can be seen in this figure, the COM distance between the micelle and drug molecules at a times of about 19 and 68 ns is gradually reduced. In addition, the less distance between micelle and drug molecules in the Micelle/ANS system indicates a stronger interaction between the carrier and ANS in this system.

## Conclusion

The orthogonal self-aggregation of two end-ornamented homopolymers and their EF-respond reversible aggregation/disaggregation through MD simulation are investigated. In the first part, the formation of the PS-β-CD/PE-FE micelle is specified via simulating the self-assembly process, and it is characterized that a stable micelle could be formed at the ns-scale. The total energy analysis is utilized to characterize the micellar system stability and interactions between the host and guest polymers. Our simulations suggest that the L–J interaction and hydrophobic effect are the driving forces for the PS-β-CD/PE-FE molecules association. Coulombic and hydrogen bonding interactions also play important roles in the PS-β-CD/PE-FE aggregation. The new micellar complex is highly sensitive to the external EF, and different EF strengths can control the self-disaggregation speed of these micelles. In the next part, through adjusting and controlling EF, the reversible assembly-disassembly transition of the micelle came true., the reversible assembly-disassembly transition of the micelle came true. Our results predicted that this type of EF-respond supramolecular assembly can act as drug transfer nano-capsules. Accordingly, the adsorption mechanism of the PS-β-CD/PE-FE micelle and two drugs, ANS and MIC, randomly distributed around the micelle is evaluated. There are a few important points in the drug adsorption process:

1. Our analysis demonstrates that PS-β-CD/PE-FE micelle as a drug carrier spontaneously encapsulates ANS and MIC molecules in water environments.
2. The investigation of the total energy between micelle and drugs assesses the adsorption drugs process as favorable.
3. The drug adsorption process is principally driven by L–J and hydrophobic interactions; Coul interactions also play important role during adsorption.

Finally, it was concluded that with the adsorption of drugs inside the micelle, the PS-β-CD/PE-FE could be successfully utilized as a promising drug carrier. The molecular dynamics simulation study provided further support to the established model and experimental results.

## Supplementary Information

Supplementary Information.

## Data Availability

Authors can confirm that all relevant data are included in the article and/or its supplementary information files.

## Code availability

The codes that support the findings of this study are available from the corresponding authors upon reasonable request.
