# Supplementary material for: Validation of an MD simulation approach for electrical field responsive micelles and their application in drug delivery
Source: Sci Rep. 2023 Feb 15;13:2665. doi: 10.1038/s41598-023-29835-y (PMC9931758; doi:10.1038/s41598-023-29835-y)
Supplement: Supplementary file 1 — Supplementary Information. [file 41598_2023_29835_MOESM1_ESM.docx]

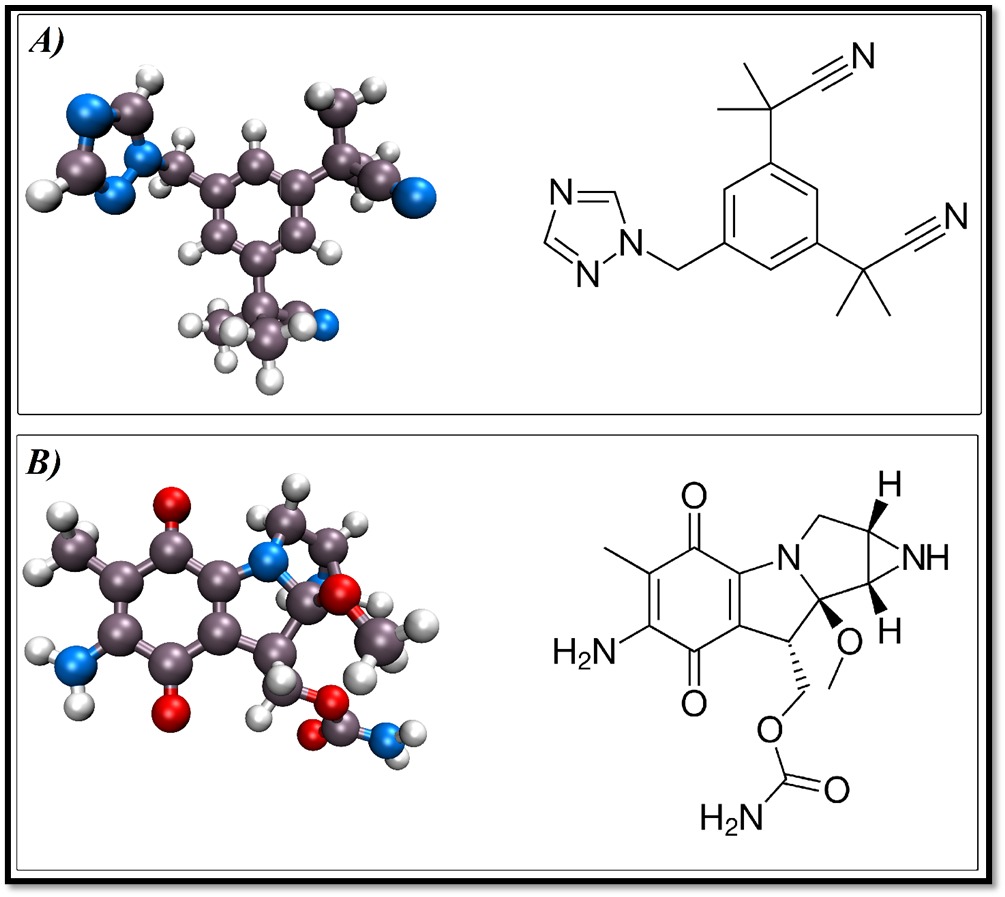


**Figure S1.** Balk-stick representation and chemical structure of A) Anastrozole and B) Mitomycin C molecules.


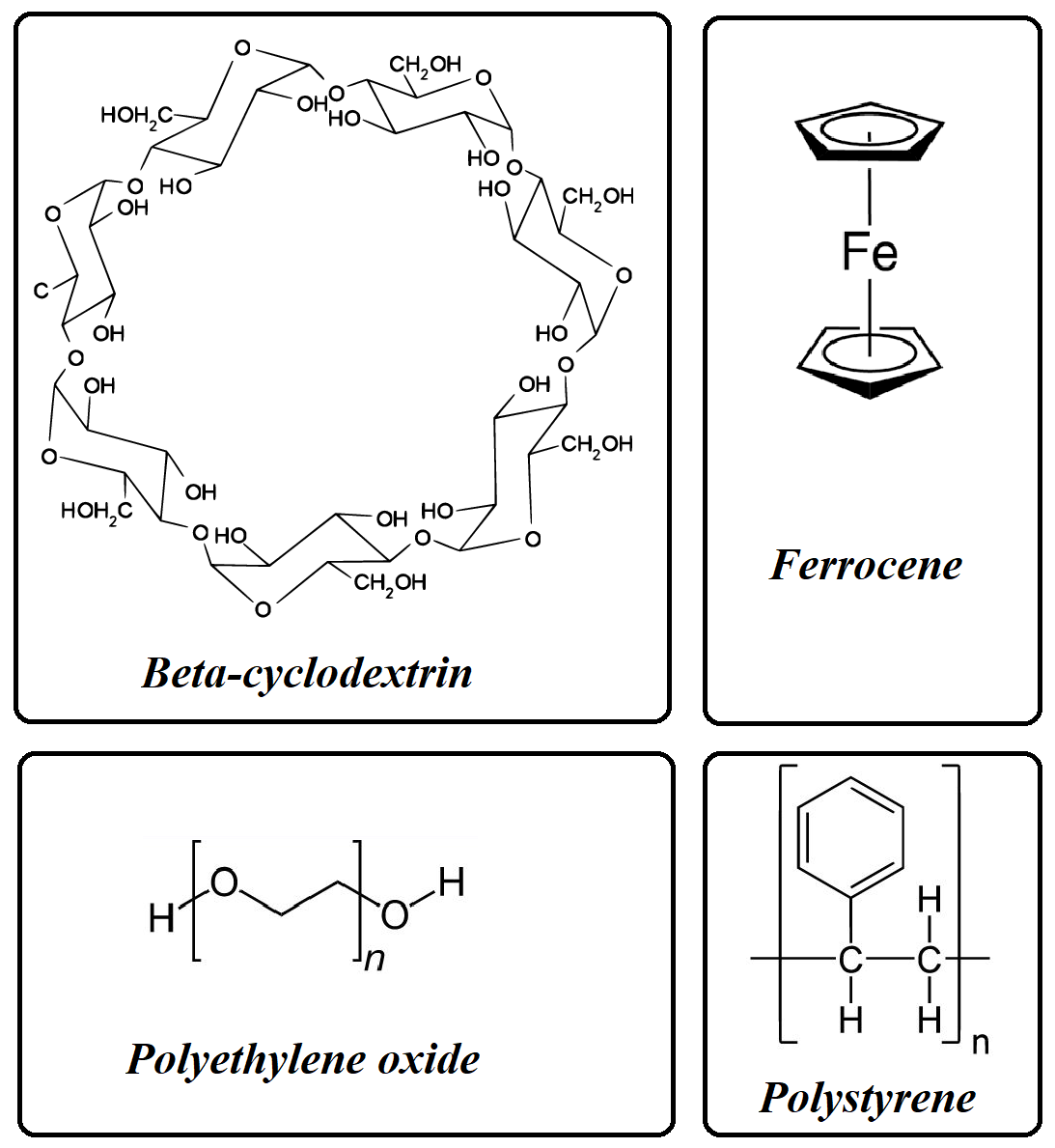


**Figure S2.** Chemical structure of the PS-β-CD/PE-FE micelle components.


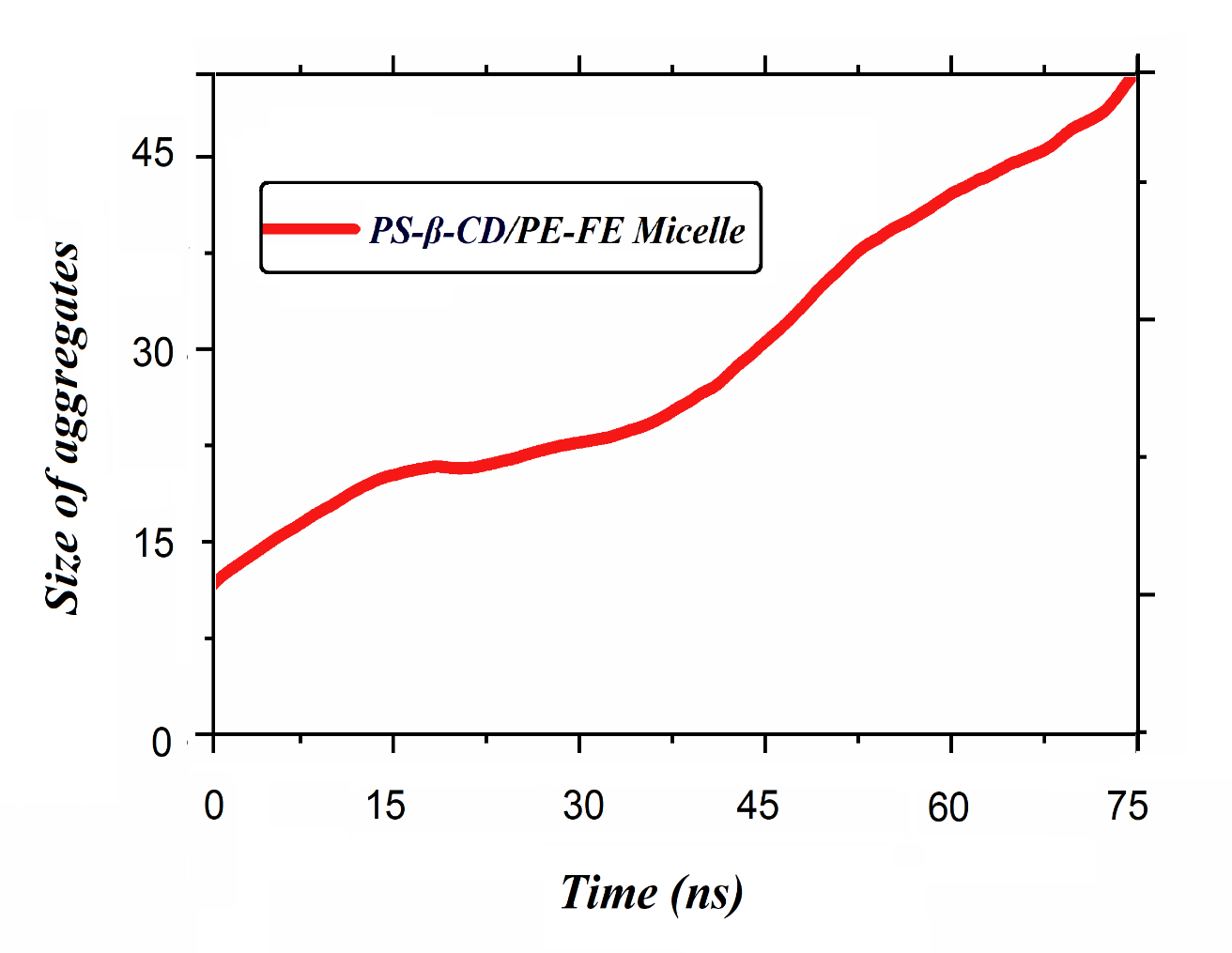


**Figure S3.** Size of the aggregate in micelle formation process.


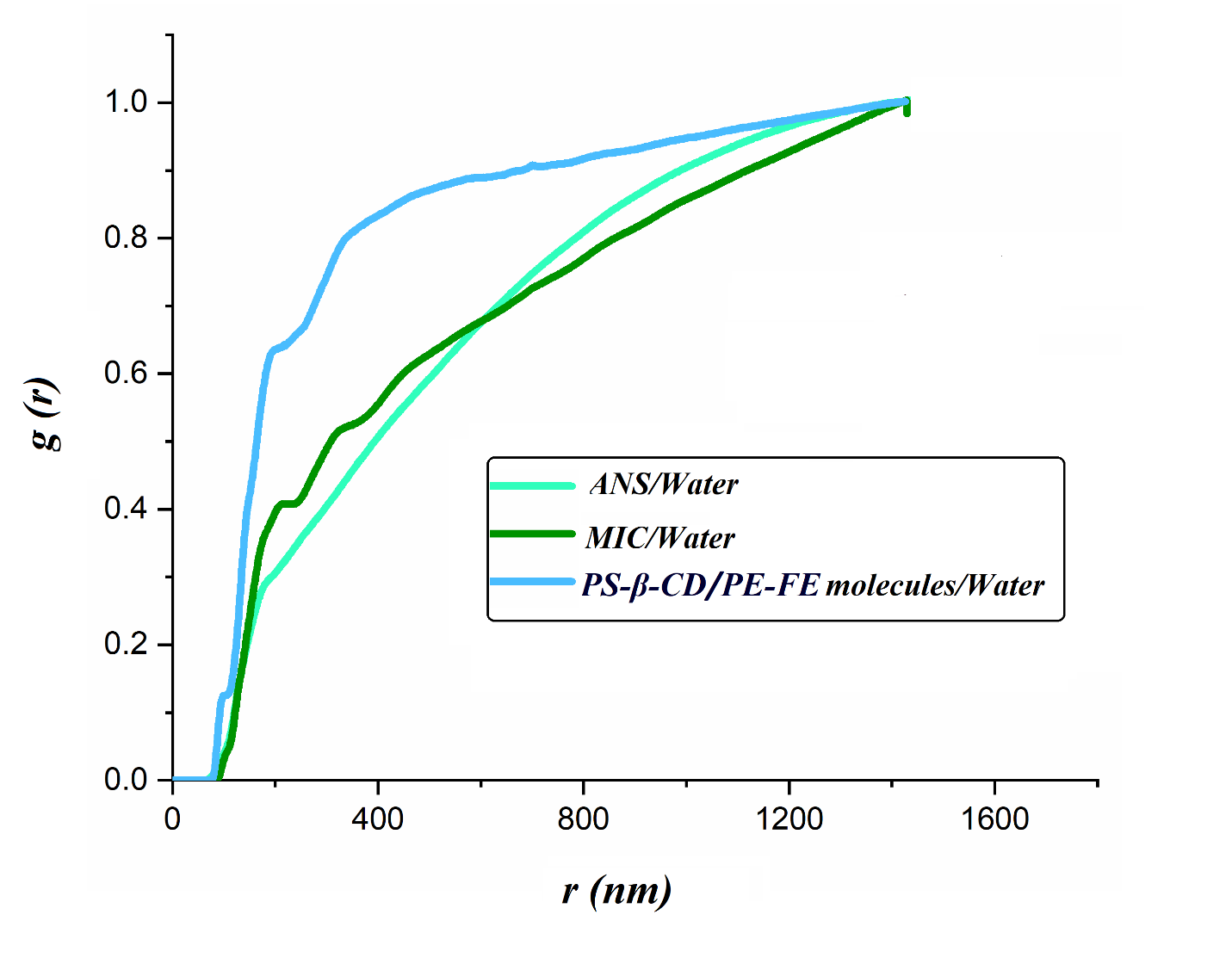


**Figure S4.** Water molecules distribution around of micelle and drugs molecules.

**Table S1.** Details of the anti-cancer drugs studied.

| **Drug** | **PubChem CID** | **Biopharmaceutical classification system drug classification** | **Bioavailability** | **Ref** |
| --- | --- | --- | --- | --- |
| ANS | 2187 | III, Adequate solubility and poor permeability | High | 7-9 |
| MIC | 5746 | II, Poor solubility and high permeability | Poor | 12-14 |

**Table S2.** The systems under electric field used in our stu­­­­­­­dy.

| **Systems** | **­­­­­­Description** |
| --- | --- |
| Micelle (+1.5) | The Micelle system with applying an EF with electric field strengths +1.5 V /nm. |
| Micelle (+3) | The Micelle system with applying an EF with electric field strengths +3 V/nm. |
| Micelle (-3) | The Micelle system with applying a negative EF -3 V/nm. |

**Table S3:** Details of studied simulation boxes.

| System | Carrier | No.  Drugs | No. Water molecules | No.  Na^+^ | No.  Cl^-^ | Box size  (nm^3^) |
| --- | --- | --- | --- | --- | --- | --- |
| Micelle | - | - | 45672 | 44 | 44 | $6\times6\times9$ |
| Micelle (+1.5) | - | - | 45672 | 44 | 44 | 6$\times$6$\times9$ |
| Micelle (+3) | - | - | 45672 | 44 | 44 | 6 $\times$6 $\times9$ |
| Micelle (-3) | - | - | 45672 | 44 | 44 | 6 $\times$6$\times9$ |
| Micelle/ANS | PS-β-CD/PE-FE | 5 | 9804 | 29 | 29 | 6 $\times6\times9$ |
| Micelle/MIC | PS-β-CD/PE-FE | 5 | 9796 | 29 | 29 | 6 $\times6 \times9$ |

**Table S4.** The obtained pair interaction energy between PS-β-CD/PE-FE molecules

and water molecules of the investigated systems at the first (0 ns) and the last (75

ns) of the MD production (All energy values are in kJ/mol).

|  | L-J | | Coul | |
| --- | --- | --- | --- | --- |
| Time | **0 ns** | **75ns** | **0 ns** | **75 ns** |
| Micelle | -5160.04 | -2015.45 | -5051.19 | -4241.78 |
| Micelle (+1.5) | -4770.89 | -2484.75 | -4154.54 | -4475.17 |
| Micelle (+3) | -2211.91 | -5160.04 | -4133.45 | -4528.71 |
| Micelle (-3) | -2484.75 | -1886.13 | -4528.71 | -4241.74 |
